# Darwin’s naturalization hypothesis does not explain the spread of nonnative weed species naturalized in México

**DOI:** 10.7717/peerj.5444

**Published:** 2018-08-17

**Authors:** Judith Sánchez-Blanco, Ernesto V. Vega-Peña, Francisco J. Espinosa-García

**Affiliations:** 1 Instituto de Investigaciones en Ecosistemas y Sustentabilidad, Universidad Nacional Autónoma de México, Morelia, Michoacán, México; 2 Posgrado en Ciencias Biológicas, Universidad Nacional Autónoma de México, Ciudad de México, México

**Keywords:** Invasive species, Risk analysis for naturalized species, Biotic interactions in plant invasions, Nonnative species residence time, Global biotic change

## Abstract

**Background:**

Despite numerous tests of Darwin’s naturalization hypothesis (DNH) evidence for its support or rejection is still contradictory. We tested a DNH derived prediction stating that nonnative species (NNS) without native congeneric relatives (NCR) will spread to a greater number of localities than species with close relatives in the new range. This test controlled the effect of residence time (*Rt*) on the spread of NNS and used naturalized species beyond their lag phase to avoid the effect of stochastic events in the establishment and the lag phases that could obscure the NCR effects on NNS.

**Methods:**

We compared the number of localities (spread) occupied by NNS with and without NCR using 13,977 herbarium records for 305 NNS of weeds. We regressed the number of localities occupied by NNS *versus Rt* to determine the effect of time on the spread of NNS. Then, we selected the species with *Rt* greater than the expected span of the lag phase, whose residuals were above and below the regression confidence limits; these NNS were classified as widespread (those occupying more localities than expected by *Rt*) and limited-spread (those occupying fewer localities than expected). These sets were again subclassified into two groups: NNS with and without NCR at the genus level. The number of NNS with and without NCR was compared using *χ*^2^ tests and Spearman correlations between the residuals and the number of relatives. Then, we grouped the NNS using 34 biological attributes and five usages to identify the groups’ possible associations with spread and to test DNH. To identify species groups, we performed a nonmetric multidimensional scaling (NMDS) analysis and evaluated the influences of the number of relatives, localities, herbarium specimens, *Rt*, and residuals of regression. The Spearman correlation and the Mann–Whitney *U* test were used to determine if the DNH prediction was met. Additionally, we used the clustering objects on subsets of attributes (COSA) method to identify possible syndromes (sets of biological attributes and usages) associated to four groups of NNS useful to test DNH (those with and without NCR and those in more and fewer localities than expected by *Rt*).

**Results:**

Residence time explained 33% of the variation in localities occupied by nonnative trees and shrubs and 46% of the variation for herbs and subshrubs. The residuals of the regression for NNS were not associated with the number or presence of NCR. In each of the NMDS groups, the number of localities occupied by NNS with and without NCR did not significantly differ. The COSA analysis detected that only NNS with NCR in more and fewer localities than expected share biological attributes and usages, but they differ in their relative importance.

**Discussion:**

Our results suggest that DNH does not explain the spread of naturalized species in a highly heterogeneous country. Thus, the presence of NCR is not a useful characteristic in risk analyses for naturalized NNS.

## Introduction

Darwin’s naturalization hypothesis (DNH), also known as the “phylogenetic repulsion” hypothesis (Mack, 1996b; Daehler, 2001; Duncan & Williams, 2002; Strauss, Lau & Carroll, 2006; Procheş et al., 2008), proposes that the phylogenetic closeness between two species impedes their coexistence within the same community because they share similar resource requirements, resulting in intense competition between them; in addition, the natural enemies of the native species would attack the nonnative, again hindering species coexistence.

DNH has been tested several times, however, the evidence for its support (Mack, 1996a; Rejmánek, 1996b; Rejmánek, 1999; Ricciardi & Atkinson, 2004; Strauss, Webb & Salamin, 2006; Schaefer et al., 2011; Bezeng et al., 2015) or rejection (Duncan & Williams, 2002; Lambdon & Hulme, 2006; Küster et al., 2008; Diez et al., 2009; Park & Potter, 2013; Li et al., 2015; Park & Potter, 2015) is still contradictory. A recent meta-analysis showed that DNH may be relevant depending on the spatial scale and the invasion stage at which the hypothesis was tested (Ma et al., 2016). The general trend, in terms of plant invasion success, was that DNH was supported at the local scale (10 × 10 m) but not at the regional scale (large territories, such as California); regarding the invasion stage (impact), DNH was not supported at the local scale, but it was supported at the regional scale. Other possible sources of confusion are the different metrics used to measure kinship relationships and the degree of invasiveness of the species considered when testing DNH (Thuiller et al., 2010).

However, even using the same spatial scale and metrics to measure kinship, contradictions persist. For example, in California, highly invasive grasses in natural environments were phylogenetically more distant to the native grasses than to the less invasive grasses (Strauss, Webb & Salamin, 2006), but in the tribe Cardueae (Asteraceae), the NNS that were phylogenetically more closely related to native species were more likely to become invasive than noninvasive (Park & Potter, 2013). A high likelihood of becoming invasive among NNS with close NCR was confirmed in the Asteraceae family in countries with Mediterranean climates (Park & Potter, 2015). These opposing results for Poaceae and Asteraceae might be explained if the functionality of DNH depends on taxonomic groups and/or species groups with shared functional attributes.

When estimating kinship relatedness using taxonomy at the genus scale, DNH has been supported in five regional floras in the USA (Mack, 1996a), in naturalized species of Poaceae in California, in Asteraceae, Brassicaceae (Rejmánek, 1996b; Rejmánek, 1999) and some other families in Australia (Rejmánek, 1999), and in aquatic environments (Ricciardi & Atkinson, 2004). However, DNH was not supported in the flora of New Zealand (Duncan & Williams, 2002) or in some plant families in New Zealand and Australia (Diez et al., 2009). Additionally, the invasiveness of ornamental plants was not associated with the presence of native congeners (Pemberton & Liu, 2009). Using different scales (region-habitat) and levels of taxonomic kinship in the Mediterranean Basin, no evidence was found for the influence of natives on nonnatives except in the case of rare NNS that were positively correlated with native species in the same genus (Lambdon & Hulme, 2006). Finally, the number of native congeners in Kentucky did not affect the likelihood of naturalization and invasion in NNS, but DNH was relevant at the establishment stage (Pellock et al., 2013).

Studies performed at different spatial scales also yield contradictions to the trends found in the meta-analysis (Ma et al., 2016). For example, DNH is supported among trees and shrubs in three countries in southern Africa (Bezeng et al., 2015) and the Azores island flora (Schaefer et al., 2011); however, DNH was not supported in the German neophyte flora (Küster et al., 2008). When the invasion process was followed for more than 40 years in 480 plots of 0.5 × 2.0 m in New Jersey, USA, the NNS with close native relatives became dominant, and the native relatives went locally extinct, again contradicting DNH (Li et al., 2015). Even within the same community, the results are contradictory when using phylogenetic dispersion at different scales: DNH is supported at a fine scale but is not at a large scale (Carboni et al., 2013). Thus, there is still no clear pattern of phylogenetic repulsion that can be used in predicting which species will become invasive. However, determining how, where and when DNH is functional is important in predicting which exotic species will be problematic.

The invasion process has rarely been considered in tests of DNH. In the meta-analysis by Ma et al. (2016), the final stage, “impact”, was addressed, but the “establishment” and “lag” phases (the latter of which is defined as the period that occurs after an NNS becomes established in a new area until it forms new populations) were not adequately considered in those tests. In the lag phase, evolutionary processes occur that depend on several biological, environmental and anthropic factors that interact with one another and determine the stabilization and distribution of NNS (Wilson et al., 2007). After some time, this process makes possible the explosive expansion of some species that become invasive, which is known as the “expansive” or “exponential” phase (Williamson, 1996; Crooks, 2005).

Additionally, residence time has been ignored in the analysis of the invasion process (Wilson et al., 2007); this variable integrates a suite of factors, such as the probability of naturalization, the extent of the occupied area and the overcoming of the lag phase (Richardson & Pyšek, 2006). The residence time usually explains up to 44% of the variation in the number of locations occupied by NNS within habitats that are suitable for their growth and reproduction (Wu, Chaw & Rejmánek, 2003; Ahern et al., 2010; Sánchez-Blanco et al., 2012).

We asked whether the control of variables that affect the spread of NNS, such as residence time and the lag phase of the invasion process, would allow a robust test of DNH considering the number of localities occupied by NNS at the regional scale. We also asked whether DNH operates only for specific functional or taxonomic groups. This possibility would explain why a test of DNH including numerous species would generate inconclusive results for that test, as the supporting results produced by one group of species would be nullified by the unsupportive results for another group of species.

In this study, DNH was tested by integrating four factors that influence the spread of NNS in the recipient area. The first is residence time, whose importance has been ignored (Wilson et al., 2007) when testing DNH and which partially determines the area occupied by introduced species. The more time an NNS has been in a new territory, the more likely it is to establish, naturalize and become an invader (Rejmánek, 2000; Castro et al., 2005; Pyšek & Jarošík, 2005; Richardson & Pyšek, 2006; Wilson et al., 2007; Pyšek et al., 2008; Ahern et al., 2010; Pyšek et al., 2015). The second factor is the stage in the invasion process, which is considered by including species that have overcome their lag phase. By using these species, we expected a reduction in the noise of the environmental and neutral filtering that can obscure or nullify the drivers of DNH during the establishment and lag phases (Li et al., 2015). The lag phase lasts from 24 to 54 years for herbs and grasses, 131 years for shrubs, 30–50 years for trees in the tropics, and 80–350 years for trees in other climes (Mack, 1991; Kowarick, 1995; Groves, 2006; Aikio, Duncan & Hulme, 2010; Crooks, 2005).

After we selected species that have overcome their lag phase, we considered the NNS occupying more or fewer localities than expected based on their residence time, which was calculated using a regression between the number of localities and residence time (obtained from the years elapsed since their first record in herbarium). In agreement with DNH, we expected that the NNS in more localities than expected would have no congeneric relatives, whereas those occupying fewer localities should have congeneric relatives. We tested this prediction first using the species that overcame their lag phase, which can be considered naturalized (Richardson et al., 2000).

The third type of factors integrated into this study was biological attributes that can affect the spread of NNS. For example life form, cycle life, grow form, fruit or propagule size, dispersal mode, fruit type, breeding system, among others (Pyšek & Richardson, 2007). We used only biological attributes that have been used as aids in risk analysis to predict which species will become invasive excluding those that require laboratory or field observations of live plants (Rejmánek & Richardson, 1996; Goodwin, McAllister & Fahrig, 1999; Kolar & Lodge, 2001). These biological attributes have been used also to determine which established or naturalized NNS could have the most severe impacts in the new range and thus, set attention priorities (Leung et al., 2002).

The fourth type of factor was the usage of NNS, which foster their introduction to different places influencing their propagule pressure in the new area (Mack, 1991; Mack, 2003). Usage has been related to the increase in propagule pressure; the more useful is a nonnative plant the higher is its introduction frequency with a high number of propagules in each event. A high propagule pressure facilitates establishment, naturalization and invasion success of NNS (Rouget & Richardson, 2003; Lockwood, Cassey & Blackburn, 2005; Colautti, Grigorovich & MacIsaac, 2006; Dehnen-Schmutz et al., 2007; Catford, Jansson & Nilsson, 2009; Lockwood, Cassey & Blackburn, 2009; Pemberton & Liu, 2009).

As kinship relationships could be acting in concert with sets of biological attributes and usages (which we call “syndromes” in this study), we also tested DNH in groups of NNS characterized by specific syndromes. We expected to find syndromes associated with NNS with and without relatives occupying more or fewer localities than expected due to residence time. We sought to identify these syndromes in NNS with residence times greater than 54 years, in which their lag phase would have ended. The syndromes were identified by grouping the species based on their attributes and usages using multivariate techniques (NMDS and COSA, see ‘Methods’) to later determine whether the groups were associated with the residuals of the regression of residence time and the number of occupied localities.

## Materials and Methods

We used a taxonomic estimates of kinship relationships. This approach has been criticized as subjective because it can bias the results when compared with estimates of phylogenetic distance (Procheş et al., 2008; Thuiller et al., 2010) or discount the evolutionary similarities among species (Lambdon & Hulme, 2006). However, taxonomic estimation is the approach used most often for identifying kinship relationships because there are no complete phylogenies for all plants at the genus level.

### Selection of the species

The analyzed nonnative and native weed species were selected from lists of the families Amaranthaceae, Asteraceae, Brassicaceae, Caryophyllaceae, Casuarinaceae, Cucurbitaceae, Cyperaceae, Euphorbiaceae, Fabaceae, Phyllanthaceae, Plantaginaceae, Poaceae, and Polygonaceae (Villaseñor & Espinosa-García, 1998; Villaseñor & Espinosa-García, 2004; Dávila et al., 2006; Sánchez-Ken, Zita-Padilla & Mendoza, 2012; Villaseñor et al., 2012). The “Malezas Introducidas en México” CONABIO database of weeds (Espinosa-García et al., 2003), which contains information on 17,385 specimens of 345 NNS from all Mexico, was used. This database includes information from the 13 most important herbariums in México MEXU, CHAPA, FCME, ENCB, ANSM, BCMEX, QMEX, IEB, EBUM, IBUG, XAL, UAS, and CICY. A detailed review of the scientific names of the species was carried out to exclude synonyms and wrongly applied names using the following electronic databases: W3tropicos (http://www.tropicos.org), The Plant List (http://www.theplantlist.org/), the Integrated Taxonomic Information System (ITIS), (http://www.itis.gov) and the International Plant Names Index (IPNI) (http://www.ipni.org/). Typical varieties or subspecies were grouped into the corresponding species. The correct identification of specimens was also verified. Specimens with conflicting taxonomy and records of cultivated species were removed from the analysis. Also, repeated specimens from the same area or localities were eliminated. Thus, the specimen number decreased to 13,977 and the respective species to 305.

The year of the first record for each species was obtained from the oldest herbarium specimen; otherwise, information from the catalogs of Asteraceae (Villaseñor et al., 2012) and Brassicaceae (S Rojas-Chávez & H Vibrans, 2015, unpublished data, available upon request from H. Vibrans: http://heike@colpos.mx) was used. Historical descriptions from the Herbarium Catalog of the Royal Botanical Expedition of New Spain (Blanco y Fernández de la Caleya, Espejo & López, 2010) and the Royal Botanical Expedition to New Spain, 1787–1803 (Mociño & Sessé, 2010a; Mociño & Sessé, 2010b) were used to obtain data on species introduced to New Spain during the colonial period.

For each year of botanical activity (collection of specimens of NNS by botanists providing herbarium specimens), the number of NNS, newly detected NNS, and herbarium specimens collected were obtained to estimate the collection effort. For each species, the year of the first recorded herbarium specimen or the first published record was used to estimate the residence time. The residence time (*Rt*) is the difference between the reference year (2003) minus the year of the first herbarium record, report or observation of a species. This reference is the last year of records in the CONABIO database (Espinosa-García et al., 2003). If, for example, the first record of a species was in 1980, its residence time was calculated as being 23 years (*Rt* = 2003–*x*, where *x* is the year of the first record). The number of localities occupied by each species was obtained from the “Malezas introducidas en México” database.

### Correction of the underestimation of residence time

Residence time is underestimated by the simple fact that the year of the first record in a herbarium is not the true year of the arrival of the species (Rejmánek, 2000; Hamilton et al., 2005; Aikio, Duncan & Hulme, 2010). To correct the underestimation of the residence time, a method proposed by Espinosa-García et al. (2013) was used. First, the empirical relationship of the total number of detected species (*n*) as a function of collection effort (*ef,* number of herbarium specimens collected) for each year was calculated {*n* = (0.7402232 × *ef*)∕(1 + 0.0031367 × *ef*); *R*^2^ = 0.98}. This model shows that the maximal number of new NNS that can be detected in one year is 200 (*n*_*max*_ = 200) by collecting 1,800 or more herbarium specimens. The *n* values were rescaled (*ns* = *n*∕200; 0 ≤*ns* ≤ 1) so the *ns* proportions can be considered to be detection probabilities (*dp*) as a function of *ef*. If so, the probability of remaining undetected *(pu)* is: *pu* = 1–*dp*. As the collection effort is known for each year, it is possible to calculate the corresponding *dp* and *pu* for that year. The adjusted residence time for species *i* is defined as *art*_*i*_ = *rt*_*i*_ + (100∗*pu*_*i*_), where *pu*_*i*_ is the probability of remaining undetected in the year when species *i* was collected, and *rt*_*i*_ is the residence time (defined above) of that species.

This model identifies the detection probability for a new NNS as a function of the collection effort per year (total number of herbarium specimens of NNS); it then calculates the number of years to be added to the initial record of the NNS to compensate for the underestimation of its residence time.

### Spread and kinship relationships

**Figure 1:**
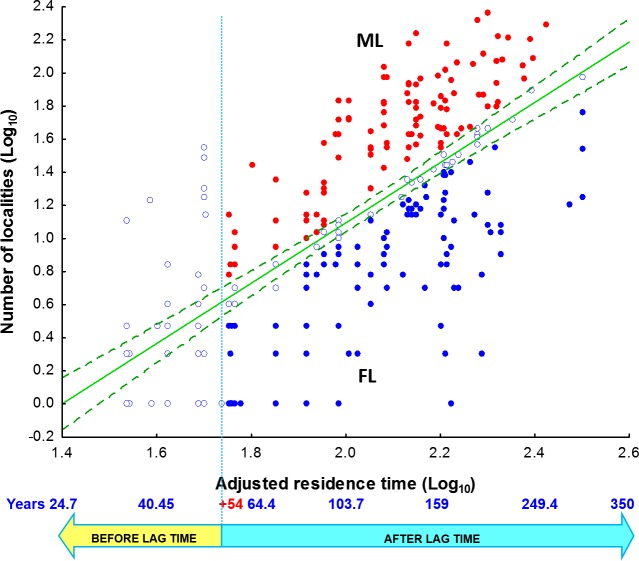
Number of localities per nonnative species vs. residence time of herbs and subshrubs species. The vertical dotted line marks the limit of the “lag phase”; the green dotted lines limit the regression confidence intervals (for 95% confidence). ML = section of the graph with species that passed their lag phase with more localities than the expected according to their residence time (full red dots). FL = section of the graph with species that passed their lag phase with fewer localities than the expected according to their residence time (full blue dots).

Two groups of NNS were chosen for the analysis based on their life form, herbs and subshrubs (*n* = 278), and trees and shrubs (*n* = 27), due to the difference in their lag phase duration (Tables S1 and S2). Their spread was obtained using a linear regression of the number of localities occupied by the species (log_10_ transformed) as a function of its adjusted residence time (log_10_ transformed). The position of a species above or below this line indicates whether it appears in more or fewer localities than expected by residence time (Fig. 1). If the species appears above the confidence interval (CI) of the line, then it occupies more localities than expected (it has a positive residual). If it appears below the confidence interval, then it appears in fewer localities than expected (it has a negative residual). If the species occurs within the CI, then its frequency does not differ from the model predictions. The regression analysis was performed using the statistical package Statistica, version 8.0 (StatSoft, Tulsa, OK, USA).

### Localities occupied by nonnative species and native relatives

The graph of the regression between the number of occupied localities and the adjusted residence time was divided into two areas considering the lag phase limit, and the species were grouped based on the regression confidence intervals: those occupying more localities than expected (ML) and those occupying fewer localities than predicted (FL) by the model (Fig. 1). The lag phase was surpassed if the residence time was greater than 54 years for herbs and subshrubs or 300 years for shrubs and trees. To determine whether the spread of NNS is related to the presence of NCR, we only selected species with a residence time longer than the lag phase, implying that they had become naturalized (*sensu* Richardson et al., 2000) in México. The shrubs and trees were not analyzed because the sample sizes were too low. Herbs and subshrubs were classified in a contingency table of localities (more or fewer localities than predicted by the regression model) and the presence of relatives (with and without), which was analyzed using a *χ*^2^ test. To evaluate whether the number of relatives of an NNS is related to the number of localities in which it occurs, the Spearman correlation between the numbers of native relatives and the residuals of the species from the residence time vs the number of localities model was calculated. According to DNH, the Spearman correlation should be negative, which means that an NNS found in more localities has zero relatives and that in few localities has relatives. A positive correlation would mean that NNS with more relatives would be found in more localities, and those with fewer relatives would be found in fewer localities.

### Identification of syndromes associated with species groups

To determine whether there were distinctive syndromes associated with groups of naturalized widespread and limited-spread herb and subshrub species (Fig. 1, region ML and FL), each species was characterized by a set of 50 biological attributes and usages associated with invasion potential in weed risk analysis (Data S1).

We did not use many useful biological attributes that have been used in weed risk analyses in other regions because they are not easily available or have to be assessed using observations of live plants in the field or laboratory, for example, growth rate, physiology, flower phenology, pollination mode, optimal germination period, optimal temperature interval, period of reproduction or flowering, plant height, leaf length, seed mass, seed bank, and leaf area index (Williamson & Fitter, 1996; Pyšek & Richardson, 2007; Dawson, Burslem & Hulme, 2009; Grotkopp, Erskine-Ogden & Rejmánek, 2010; Van Kleunen, Weber & Fischer, 2010). In other cases, they must be obtained from laboratory analyses, such as the size of the genome or the number of sets of chromosomes.

Fifty biological attributes and usages were selected considering their availability in the literature and/or by direct observation in herbarium specimens; they were grouped into two types: (a) biological attributes, and (b) usages. The most common usages of nonnative plant species in México are for food, erosion control or the stabilization of dunes, forage, medicinal and ornamental purposes.

A matrix with 278 rows (herb and subshrub species) and 50 columns (biological attributes and usages) was constructed to group the species (Data S1) and test DNH. Binary values were used to indicate the presence (1) or absence (0) of biological attributes and usages, and numerical values were used for the number of native relatives, localities, collection effort (number of herbarium specimens collected of NNS), residuals of the regression and residence time.

A heat map “Heatplus” package in “R” platform, (Ploner, 2014) was used to eliminate biological attributes and usages that did not contribute to the classification of NNS (Fig. S1). This technique relates the dendrograms of species based on their biological attributes and usages with the dendrogram of attributes and usages based on their incidence in the species. The Jaccard coefficient was used to construct distance matrices using the package ecodist (Goslee & Urban, 2013). Dendrograms were built using the unweighted pair group method with arithmetic mean (UPGMA) with the hclust function in the Stats package (R Core Team, 2016b). The dendrograms were drawn using the package ggplot2 (De Vries & Ripley, 2015). The number of significant groups for each dendrogram was obtained with the cophenetic coefficient to obtain the height of the cut. Eleven biological attributes and usages that did not contribute to the classification were eliminated (marked with an asterisk in Fig. S1), so the analyses were performed using the remaining 39 biological attributes and usages (Data S2).

### Identification of syndromes associated with spread to test DNH

#### NMDS analysis

To detect species groups based on their biological attributes and usages to test DNH, we performed a nonmetric multidimensional scaling (NMDS) analysis (Oksanen, 2011) using 39 biological attributes, and usages (Table 1) among 278 species of herbs and subshrubs (Data S2). The groups produced by the NMDS analysis were identified using the K-means method (Hartigan & Wong, 1979) in the package rpart.plot (Milborrow, 2016). The influences of the number of relatives, number of localities, collection effort (number of herbarium specimens of NNS), residence time and residuals of the regression on the NMDS groups were evaluated using intensive permutation methods in the function “envfit” in the package vegan (Oksanen et al., 2016) with 999 permutations (Oksanen, 2017).

To test DNH in each group, the Spearman correlation and the Mann–Whitney U test were used to determine whether the number of NCR and the number of localities occupied by the NNS, and the number of localities occupied by the NNS with and without relatives were associated as predicted by DNH.

According to the predictions of DNH, (a) a negative correlation is expected between NCR at the genus level and the number of localities occupied by NNS, and (b) the number of localities occupied by species without relatives would be higher than that occupied by species with relatives.

**Table 1:** Biological attributes and usages of nonnative species of herbs and subshrubs used. In the NMDS (a) and clustering objects on subsets of attributes COSA analyses (b).

Attributes	Abbreviations	Meaning	Analysis
**(a) Biological attributes**			
^*^Life cycle	A	Annual	ab
	P	Perennial	ab
	AB	Annual-Biannual	ab
	AP	Annual-Perennial	ab
^*^Life form	HE	Herbs	ab
	VH	Vine and climbing herbs	ab
^*^Classes of Angiosperms	MO	Monocotyledons	ab
	DI	Dicotyledons	a
^**^Growth forms	GEO	Geophythe	ab
	HEM	Hemicryptophyte	ab
	THE	Therophyte	ab
^*^Type of fruit and dehiscence	DE	Dehiscent fruits	a
	ID	Indehiscent fruits	ab
	DF	Dry fruits	ab
	AC	Achene	a
	CA	Capsule	ab
	CR	Caryopsis	ab
	CY	Cypsela	ab
	LE	Legume or pod	ab
	SI	Siliqua or silique	ab
^***^Dispersal syndrome	AN	Anemochory	ab
	AU	Autochory	ab
	BA	Barochory	a
	HY	Hidrochory	ab
	ZO	Zoochory (Endozoochory, Exozoochory or Epizoochory)	ab
Dispersal distance	N	Near: behind or very close of the source plant as in barochory	ab
	AV	Average or medium: few meters away from source plant as in autochory	ab
	H	High: dozens or hundreds of meters away for source plants as in anemochory, hidrochory and zoochory	ab
	MU	Multiple: More than two dispersal syndromes	ab
^*^Size of the dispersal propagule (fruit and seed)	VS	Very small: 0–1.5 mm	ab
	S	Small: 1.6–3.5 mm	ab
	M	Medium: 3.6–7.5 mm	ab
	L	Large: 7.6–49 mm	ab
	VL	Very large: 50–1,000 mm	ab
**(b) Usages**			
^*^Usage	COM	Comestible or food	ab
	ER	Erosion or the stabilization of dunes	ab
	FO	Forage	ab
	ME	Medicinal	ab
	OR	Ornament	ab

**Notes.**

* Electronic databases: (Flora of North America Editorial Committee and eds., 1993+), (Davidse, Sousa & Chater, 1994+), (eFloras, 2008), (Vibrans, 2004–2009), International Legume Database & Information Service (ILDIS, 2005), GrassBase-The Online World Grass Flora (Clayton et al., 2006+) and (USDA-NRCS, 2015); Flora Fanerogmica del Valle de México (Rzedowski & Rzedowski, 2005); weed catalogs: Asteraceae (Villaseñor et al., 2012) and Brassicaceae (S Rojas-Chávez & H Vibrans, 2015, unpublished data) and specific monographs.

** (Raunkiaer, 1934).

*** (Dansereau & Lems, 1957; Van der Pijl, 1982) and descriptions and images of fruit and seed morphology in (Flora of North America Editorial Committee and eds., 1993+; eFloras, 2008; Vibrans, 2004–2009; USDA-NRCS, 2015; Villaseñor et al., 2012).

#### COSA analysis

To determine whether there were sets of biological attributes and usages (Data S2) characterizing groups of NNS (with or without close NCR) in more or fewer localities than expected (Fig. 1, region ML and FL), COSA (clustering objects on subsets of attributes) analysis was used. This analysis is appropriate when the differentiation among groups of objects is unclear and when there are objects that do not clearly belong to any of the groups (Kampert, Meulman & Friedman, 2016). It is a method involving iterations that minimize the distance among individuals. The measure used is the inverse exponential distance (Friedman & Meulman, 2004), which includes a parameter (lambda) that represents the closeness among objects (species in this case). Through the modification of this parameter and the size of the groups, the values of average distances within and among groups can be calculated.

The resulting COSA matrix of dissimilarity among objects was used to characterize our groups (clusters) based on the relative importance of subsets of attributes. COSA evaluates *I*_*kl*_, the importance of attribute *k* in cluster *l* (*C*_*l*_) as inversely proportional to the dispersion, *S*_*kl*_, of the data in attribute *k* for objects in cluster *l* (*C*_*l*_) Eq. (1):

(1) \begin{eqnarray*}{S}_{kl}= \frac{1}{{N}^{2}} \sum _{i,j\epsilon {C}_{l}}{d}_{ijk}\alpha {I}_{kl}^{-1}\end{eqnarray*}

where *d*_*ijk*_ is the dissimilarity between objects *i* and *j* as evaluated for attribute *k*, and *N* is the number of objects. This method was implemented using the software rCOSA (Kampert, Meulman & Friedman, 2016). The NNS were grouped *a priori* based on two criteria: occurrence in more/fewer localities and the presence/absence of NCR. The biological attributes and usages were graphed by group according to their average relative importance value. According to the predictions of DNH, distinctive individual biological attributes and usages or syndromes are expected to distinctively characterize the a *priori*-formed groups. All analyses were performed using the platform R (R Core Team, 2016a).

## Results

We obtained a significant regression model (*R*^2^ = 0.4601, *P* < 2.2e−16) between the number of localities occupied by nonnative species (*N*) and their adjusted residence time (*Rt*) (*N* =  − 2.549 + 1.8213∗*Rt*). Residence time (*Rt*) explains the 46% of the variation in the localities occupied by NNS of herbs and subshrubs (Fig. 1), while for trees and shrubs, the regression model (*N* =  − 1.626 + 1.3352∗*Rt*) was marginally significant (*R*^2^ = 0.3327, *P* = 0.001634); *Rt* explains 33.2% of the variation, but this relationship was only marginally significant*.*

### Occupied localities and the presence of native relatives of herbs and subshrubs

Eighty-seven percent of species had passed their lag phase (*n* = 243). The species occupying more (109) or fewer localities (101) than the predicted by the regression model and their confidence limits were denominated as “present in more localities (ML) than expected by residence time” and “present in fewer localities (FL) than expected by residence time” respectively (Fig. 1). The number of species with relatives in more localities than expected (57) did not differ from the number of species without relatives (52) (*χ*^2^ = 0.27103, df 1, *P* = 0.3974). The number of species with relatives in fewer localities than expected (45) did not differ from the number of species without relatives (56) (*χ*^2^ = 1.3925, df 1, *P* = 0.7742).

The number of congeneric relatives was not correlated with the residuals of the regression of residence time and localities occupied by species. Spearman’s correlation coefficients were not significant for 57 and 45 species of herbs and subshrubs, respectively (*R* =  − 0.0336, *P* = 0.7286 and *R* =  − 0.0365, *P* = 0.7168). This indicates that the number of relatives of NNS does not determined their spread.

### Species groups and their link with nonnative species spread

#### Identification of syndromes

**Figure 2:**
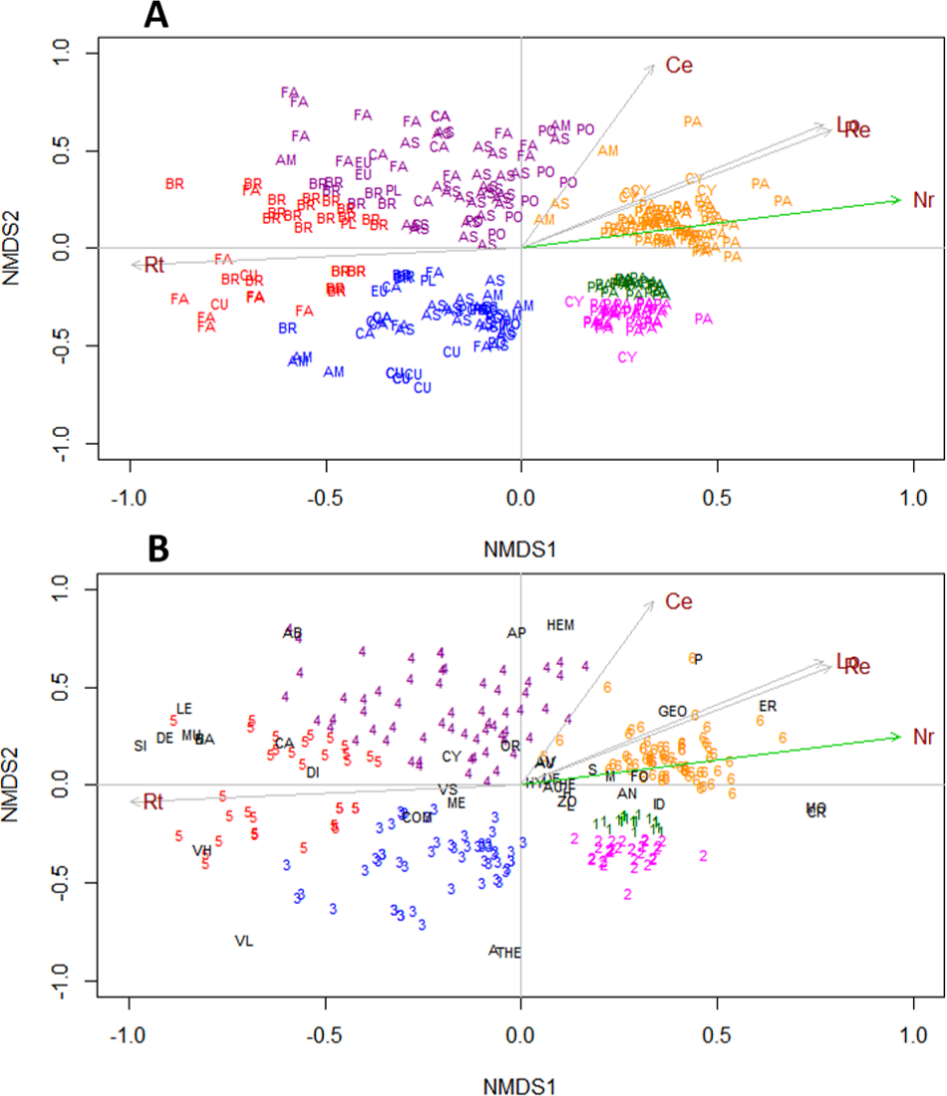
Non-metric multidimensional scaling of nonnative species of herbs and subshrubs grouped with the K-means method. (A) Families and (B) biological attributes and usages. The colors of the acronyms of the families coincide with the number of the group to which the species belong: 1 green, 2 pink, 3 blue, 4 purple, 5 red, and 6 orange. The size of the vectors represents the influence on the clustering of: Nr, number of relatives; Lo, number of localities; Hs, number of herbarium specimens; Rt, residence time and Re, residuals of the regression. The vectors Nr is significant (*P* = 0.003). Families: AM, Amaranthaceae; AS, Asteraceae; BR, Brassicaceae; CA, Caryophyllaceae; CU, Cucurbitaceae; CY, Cyperaceae; EU, Euphorbiaceae; FA, Fabaceae; PL, Plantaginaceae; PA, Poaceae and PO, Polygonaceae. See Table 1 for abbreviations.

Six groups were differentiated based on the K-means method in the NMDS analysis (*R*^2^ = 0.939, *Stress* = 0.247) according to the biological attributes and usages (Fig. 2). The permutation analysis indicated that the number of relatives (Nr) significantly influenced the formation of groups six, one and two (*P* = 0.003). Group six was characterized by grasses and sedges, perennials, and geophytes that are used as forage and erosion control or the stabilization of dunes, median dispersal distance, and have small (1.6–3.5 mm) and medium-sized (3.6–7.5 mm) propagules (Figs. 2A and 2B). The groups one and two were characterized by grass and sedge species, large propagule size (7.6–49 mm), high dispersal distance, indehiscent fruits, and dispersal through zoochory and anemochory (Figs. 2A and 2B). However, when testing DNH, the correlations within each NMDS group in terms of the number of natives species and number of localities occupied by the NNS were not statistically significant (*R*_GROUP1_ =  − 0.1805, *P* = 0.4098; *R*_GROUP2_ = 0.2877, *P* = 0.1103; *R*_GROUP3_ = 0.1003, *P* = 0.4748; *R*_GROUP4_ = 0.0636, *P* = 0.6265, *R*_GROUP5_ =  − 0.2547, *P* = 0.1398 and *R*_GROUP6_ = 0.0502, *P* = 0.6710). The Mann–Whitney *U* test was also not significant (*P* > 0.05) within all NMDS groups when comparing the number of localities occupied by species with and without NCR (*U*_GROUP1_ = 44.0, *P* = 0.4227; *U*_GROUP2_ = 73.5, *P* = 0.0956; *U*_GROUP3_ = 280.5, *P* = 0.3637; *U*_GROUP4_ = 420, *P* = 0.5433; *U*_GROUP5_ = 80.5, *P* = 0.1042 and *U*_GROUP6_ = 635.5, *P* = 0.9245). Widespread and limited-spread species were found in all groups identified in the NMDS analysis; therefore, DNH does not explain the spread of NNS.

#### Syndromes associated with species spread

**Figure 3:**
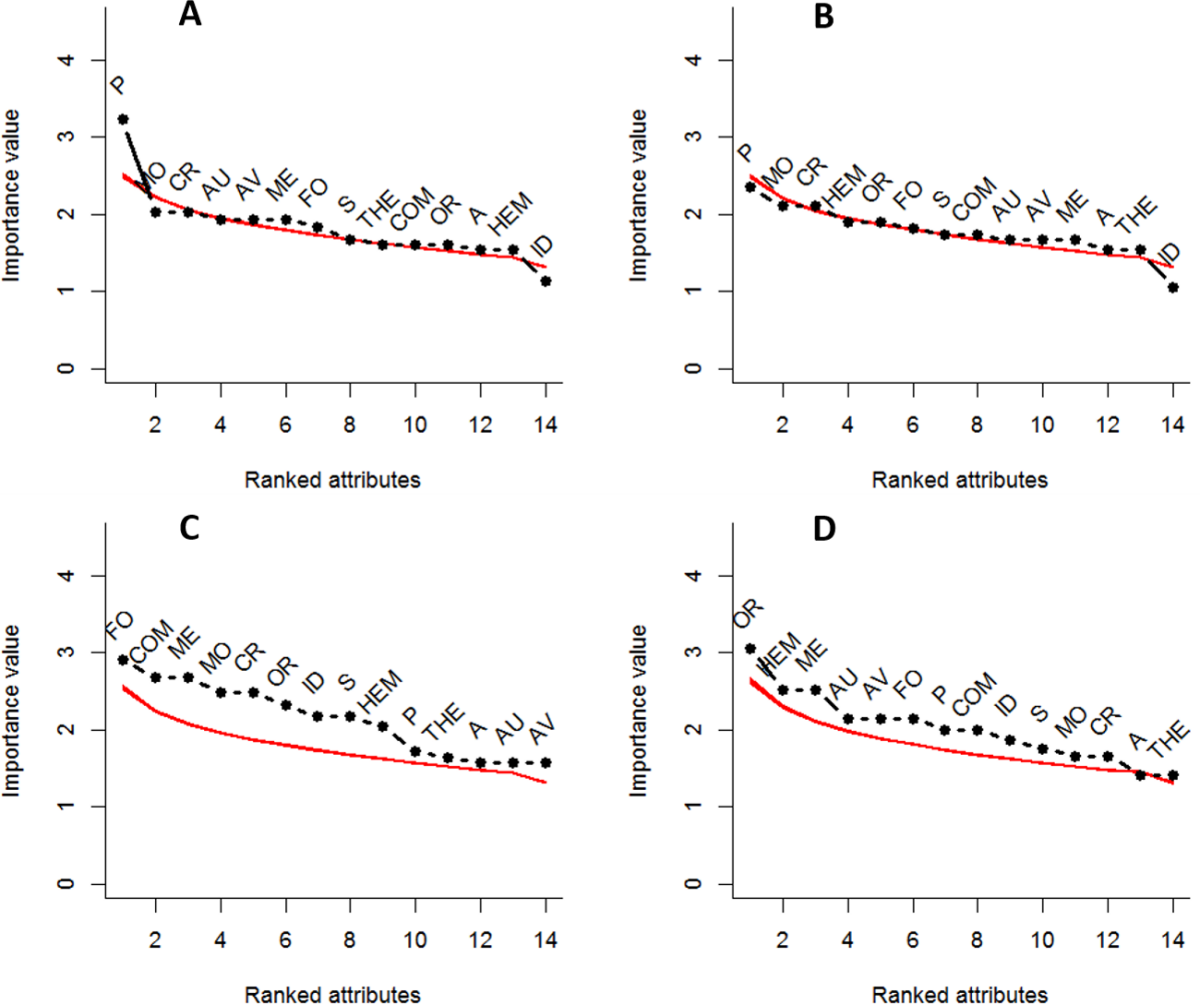
Biological attributes and usages ranked by the values of average relative importance obtained with COSA analysis, in groups of nonnative species of herbs and subshurbs. (A) Widespread species (occupying more localities than the expected by residence time) without native relatives; (B) limited spread species (occupying fewer localities than the expected by residence time) without native relatives; (C) widespread species with native relatives and (D) limited spread species with native relatives. The red line shows the values of average importance obtained from 1000 randomizations. The black dotted line shows the observed values of average importance. If observed value is above the red line, it indicates that the value is different from that observed at random. See Table 1 for abbreviations.

There were no distinctive syndromes of biological attributes and usages characterizing the groups of species without relatives occupying more or fewer localities (Figs. 3A and 3B) than expected. However, the groups of species with relatives were characterized by distinctive syndromes for more (Fig. 3C) and fewer occupied localities (Fig. 3D) than expected; these two syndromes differ in terms of the average relative importance of the biological attributes and usages (Figs. 3C and 3D). The biological attributes and usages with the highest average relative importance in the syndrome characterizing the species in more localities were in decreasing order: uses as forage, comestible and medicinal, monocotyledons with caryopsis, ornamental use, indehiscent fruits, small propagules (1.6–3.5 mm) and hemicryptophyte habit (Fig. 3C). The syndrome of the species in fewer localities was characterized by ornamental use, hemicryptophyte habit, medicinal use, autochory, forage use, perennial habit, comestible use, indehiscent fruits and small propagules (1.6–3.5 mm) (Fig. 3D).

## Discussion

Our results contradict DNH because the kinship relations were not associated with the spread of naturalized species with residence times longer than their expected lag phase length. The expectation of differential spread between NNS with and without NCR was also not met in the whole set of NNS or within the groups of species obtained by multivariate methods.

Some studies that agree with DNH used large scales and taxonomic closeness between native and NNS at the genus level as we did in our study (Mack, 1996a; Rejmánek, 1996b; Rejmánek, 1999). However, in those studies, DNH was assessed only using the presence/absence of congeneric relatives without considering, as we did, the effect of residence time, the passage of the lag phase, the biological attributes, and usages. Another important difference between those studies and ours is México’s high heterogeneity, which is reflected in its high floristic diversity (with 22,969 species), and its physiographic and biogeographic complexity (Cervantes-Zamora et al., 1990; Morrone, 2005; Villaseñor, 2016), which has no parallel in the sites used to test DNH with taxonomic closeness.

None of the studies that have tested DNH and controlled for the residence time considered the number of localities in which the NNS are present (after controlling for residence time) as a proxy for the success or failure of expansion or used naturalized species beyond their lag phase. By controlling for these variables, we assumed that we could reduce the noise of the environmental and neutral filtering that can obscure or nullify the drivers of DNH during the establishment and lag phases (Li et al., 2015). Thus, we believe that we obtained a more robust test of DNH than those performed using NNS presence/absence. The effect of NCR, if any, should be clearer after the lag phase for the species occupying more or fewer localities than expected only based on the effect of residence time. Our results indicate that the presence of NCR in a highly heterogeneous country does not hinder the spread of naturalized plant species; these results agree with the general trend found for regions showing that DNH was not supported at the regional scale (Ma et al., 2016).

A possible explanation for the disagreement between our results and the evidence in favor of DNH is that México occurs mostly outside the highly plant-invaded latitudinal bands (Rejmánek, 1996a; Sax, 2001), and it also lacks the insularity that results in high susceptibility to invasions, such as in Australia. Only 2.7% of México’s flora is composed of NNS (Villaseñor & Espinosa-García, 2004), whereas 15% and 30.6% of the floras of Australia and California are composed of NNS, respectively (Rejmánek, 1996a; Ornduff, Faber & Wolf, 2003; Invasive Plants and Animals Committee , 2016). We speculate that highly invaded regions allow the detection of the predictions of DNH, whereas lightly invaded and highly heterogeneous regions will not allow for such detection. Thus, a low degree of invasion and high heterogeneity would lower the probability of an encounter between an invader and a native relative. Moreover, at the country or state scale under highly heterogeneous conditions, nonnative and native species can be present simultaneously even if they do not occur within the same community.

Contradictory results using different scales might also be resolved if DNH is relevant at the local scale (Pellock et al., 2013), and the consequences of up scaling may or may not support DNH depending on the heterogeneity of the territory at larger scales. For example, in a large territory with low heterogeneity, the consequences of DNH at the local scale will be maintained, but they will not be maintained if the heterogeneity is high. Thus, either the heterogeneity of the invaded range that we used is not appropriate for testing DNH in naturalized species, or this hypothesis is not effective at regional or country scales when their degree of invasion is low.

The coexistence between NNS and their NCR may also depend on the disturbance intensity. Even within the same community, disturbed areas with conditions that differ greatly from the natural disturbance regime exclude native species and foster invasion by NNS (Catford, Jansson & Nilsson, 2009), but in the areas with the natural disturbance regime, the native species predominate.

To answer whether DNH could operate only for specific functional or taxonomic groups, we first used NMDS to identify groups of species based on biological attributes or usage to test DNH. Then, we formed four species groups (determined by taxonomic closeness and spread) that are useful for testing DNH using COSA analysis to determine whether there are biological attributes or usage types associated with the expected effects of native species on the spread of NNS.

Nonnative species groups were found with the NMDS analysis, but the predictions of DNH were not met for any of the groups. Although we observed some correlated biological attributes that defined some groups (which coincide with taxonomic families), none of those biological attributes or usages were associated with the spread of the species. We expected the formation of groups containing mostly widespread species or groups containing mostly limited-spread species such that the groups’ defining biological attributes and usages could be used to predict the potential spread of a recently arrived species. At the same time, we expected that if DNH is correct, the grouped widespread species would have no NCR, and the grouped species with limited-spread would have NCR. None of our expectations were met; therefore, the groups found with NMDS are not useful for risk analysis, and the grouped species did not support DNH.

Although the NMDS analysis was used for grouping NNS, its grouping algorithms were not suitable for meeting our expectations. Thus, we changed the methodological approach using COSA analysis, in which the grouping was done *a priori*, asking whether the biological attributes and usages were associated with the groups.

We grouped the NNS following two criteria: whether they were found in more or fewer localities and the presence/absence of NCR. We expected that if DNH explained the spread of NNS as a function of the presence of NCR, then we would find distinctive syndromes or single traits characterizing the groups. Contrary to our expectation, no single distinctive biological attributes, usages or syndromes characterized groups of NNS without NCR occupying more or fewer localities than expected. Instead, two syndromes associated with the two groups (widespread and limited-spread species) with NCR were detected. Both syndromes share some biological attributes and usages that differ in their average relative importance for each group.

In the syndrome associated with widespread NNS with relatives, the usage types of forage, comestible, and medicinal plants (Fig. 3C) predominated, with high importance values over those of most biological attributes. For the limited-spread NNS with close relatives the ornamental and medicinal were predominant (Fig. 3D). This can be explained because most of the species used as forage belong to Poaceae and Leguminosae, which were likely intentionally introduced to México (Challenger et al., 1998; Mack, 2003). Moreover, several species of grasses have been introduced mainly as forages and secondarily as ornamentals (Beetle, 1983; Villaseñor & Espinosa-García, 2004). Eventually, many of the intentionally introduced species escaped from cultivation, becoming feral and naturalizing in México (reviewed in Espinosa-García & Villaseñor, 2017). These intentional introductions, mainly for forage, usually imply that several introduction events with a great number of propagules occurred (Kolar & Lodge, 2001; Lockwood, Cassey & Blackburn, 2005), allowing naturalization in spite of the presence of NCR. Our results coincide with those in which propagule pressure allowed the establishment of invasive species and was more important than biological traits that may interfere with the establishment and naturalization of NNS (Dawson, Burslem & Hulme, 2009).

Nonnative species have been propagated and commercialized because of their uses (Williamson, 1996; Mack, 2003; Pyšek et al., 2003), which is important in explaining and predicting nonnative establishment and/or naturalization (Mack, 1991; Kowarik, 2005; Lockwood, Cassey & Blackburn, 2005). For example, the high demand and importation of nonnative plant species over a long period increased their naturalization, favoring plant invasions (Williamson & Fitter, 1996; Pemberton & Liu, 2009). High propagule pressure enables NNS to overcome ecological filters in different stages of the invasion process, favoring naturalization and invasion; the continuous introduction of propagules allows the entry of new genetic materials, the formation of hybrids and the selection of new varieties that favor locally adapted biotypes (Wilson et al., 2007). The species introduced as a result of high propagule pressure show rapid evolution and local adaptation, which enable them to occupy a wide range of new habitats (Oduor, Leimu & Kleunen, 2016). Furthermore, useful NNS have been more invasive than those that are not useful due to assisted propagation, protection from pests and propagule pressure (Williamson, 1996).

Although some of the biological attributes that characterized the lower than expected spread group (Fig. 3D) have been reported in successful invasive species of semi-natural habitats (Pyšek & Richardson, 2007), their presence could be explained if many of these species colonized all the few suitable habitats available in the new range. The spread of these NNS in few localities does not mean that they have failed in their expansion or their “invasion”; an explanation would be that the species are found in areas where geographic barriers exist that have not allowed them to reach other suitable habitats. The explanations of the availability of few suitable habitats and the separation from suitable habitats by geographic barriers can be verified by modeling the potential distributions of these species.

Another explanation for widespread species with NCR would be Darwin’s preadaptation hypothesis (DPH), which proposes that the niche similitude between nonnatives and their NCR will enable their coexistence in the new range (Duncan & Williams, 2002; Diez et al., 2008). Our results do not support DPH because the expected pattern of widespread species with NCR was found only in one of the four groups (Fig. 3C); the other group of widespread species has no NCR (Fig. 3A). Furthermore, the expected limited-spread when NCR is absent was found in only one (Fig. 3B) of the remaining groups. Neither DNH nor DPH satisfactorily explains the spread of naturalized plants for which historical factors (introduction frequency and usage) can be more important than biological factors in determining their geographical spread (Castro et al., 2005).

We expected that syndromes were better than the sum of individual scores of biological attributes and usages in predicting which species will become invasive and which will be just additions to the local biodiversity. Although single biological attributes and usages are shared between NNS that occupy more and fewer localities than predicted, their average relative importance differs. These results suggest that syndromes favor the naturalization of species more than individual traits. However, we found that these sets of biological attributes and usages have no diagnostic value for risk analyses of naturalized species.

There is still no definite invasion syndrome (Lloret et al., 2005) nor has it been possible to find generalizations of biological attributes that determine the invasion success of exotic species; our results agree with these assertions.

DNH integrates Darwin’s ideas that were later formalized into eight hypotheses that explain the antagonistic effects of natives on nonnatives (Catford, Jansson & Nilsson, 2009), and therefore, the DNH is now firmly included in the theoretical framework of invasion ecology in spite of the contradictory results in its testing. The contradictory results of tests of DNH may be explained considering that the predictions derived from that hypothesis can be affected by many variables other than kinship, competition and the effect of natural enemies. For example, environmental neutral selection and the ever-changing environments during the invasion process may or may not favor the driving processes of DNH (Li et al., 2015).

Additionally, as we have shown, there are biological, environmental and anthropogenic factors that interact with one another (Wilson et al., 2007) and that can contribute to the understanding of the success of the invasion process, but they have not been considered in some studies because of the complexity in obtaining such information for hundreds of NNS. Studies are needed at the local or community scales in lightly invaded areas to test the effect of kinship relationships on the extent of the dispersal of naturalized NNS to determine whether it is appropriate to use DHN in risk analysis.

## Conclusions

### Theoretical implications

Darwin’s naturalization hypothesis did not explain the spread of naturalized species in a highly heterogeneous country. This implies that the intense competition between close relative species and the transfer of native enemies to the nonnative relatives as predicted by the DNH did not occur at all or with enough intensity to exclude the nonnative relatives. By focusing on naturalized species and discounting the effect of residence time, we expected to obtain a clean signal of the operation of the DNH drivers by reducing the effect of neutral filtering and the changes that occur over the invasion process. We did not detect any signal of the operation of the DNH drivers when considering all the NNS or groups of them.

Several variables could have prevented the drivers of DNH from operating: disturbance, spatial isolation among relatives in large and heterogeneous environments, and the effects of high and repeated propagule pressure, which is aided by human protection among useful NNS. The inclusion of these variables in future DNH tests should generate a clearer understanding of the operation of competition between closely related native and NNS and the effect of native natural enemies on NNS. The general pattern is that DNH is supported at the local scale (Ma et al., 2016), but tests of that hypothesis have not included mosaics of varying perturbation intensity with various propagule pressure treatments including human protection of the nonnatives. Long-term monitoring of the invasion process is also needed.

Additionally, it is necessary to know whether the mechanisms of DNH operate differentially in the invasion stages or whether they fail and whether they operate as environmental filters that leave a trace that can be detected in the late stages of the invasion process.

### Practical implications

Our results suggest that DNH is not applicable to naturalized species in México, and it would therefore not be functional in risk analyses of naturalized species at the regional scale in highly heterogeneous countries. The functionality of DNH in the early stages of the invasion process remains to be determined; it has been reported that DNH is functional in the establishment stage (Pellock et al., 2013), but we do not know of a test of DNH during the lag phase.

We found syndromes defined by sets of biological attributes and uses in some groups of NNS with relatives, which were not functional in risk analyses of naturalized species. The characteristics alone were never diagnostic of any group of NNS, so care must be taken to assign a value to a single biological attribute in risk analyses. This implies that we should evaluate the ways in which risk analyses are conducted where the independence of the characteristics is assumed when in fact they can be correlated or sets of them could be important in the prediction of the probability that an NNS will become invasive.

##  Supplemental Information

10.7717/peerj.5444/supp-1 Data S1 Database for 305 nonnative species used for the regression analysis between adjusted residence time and the number of localities occupied First worksheet: the database for 278 nonnative species of herbs and subshrubs used for NMDS and rCOSA analyses; Second worksheet: list of species and acronym; Third worksheet: the database for 27 nonnative species of trees and shrubshrubs; and Fourth worksheet: biological attributes and other features keys for all species.

10.7717/peerj.5444/supp-2 Data S2 Database of biological attributes and usages of 278 nonnative species of herbs and subshrubs used to generate a heat map First worksheet: the database for 278 nonnative species herbs and subshrubs used in the heat map; Second worksheet: list of species and acronym; Third worksheet: biological attributes and other features keys for all species.

10.7717/peerj.5444/supp-3 Figure S1 Heat map of nonnative species of herbs and subshrubs and biological attributes and usages * Biological attributes and usages eliminated (did not contribute to the classification of NNS).

10.7717/peerj.5444/supp-4 Table S1 List of nonnative species of herbs and subshrubs included in the study *= Species with residence time smaller than 54 years.

10.7717/peerj.5444/supp-5 Table S2 List of nonnative species of trees and shrubs included in the study *= Species with residence time smaller than 300 years.

## Additional Information and Declarations

### Data Availability

The following information was supplied regarding data availability: The raw data are provided as Supplemental Files.
