# Supplementary figures and images for: Darwin’s naturalization hypothesis does not explain the spread of nonnative weed species naturalized in México
Source: PeerJ. 2018 Aug 17;6:e5444. doi: 10.7717/peerj.5444 (PMC6100849; doi:10.7717/peerj.5444)

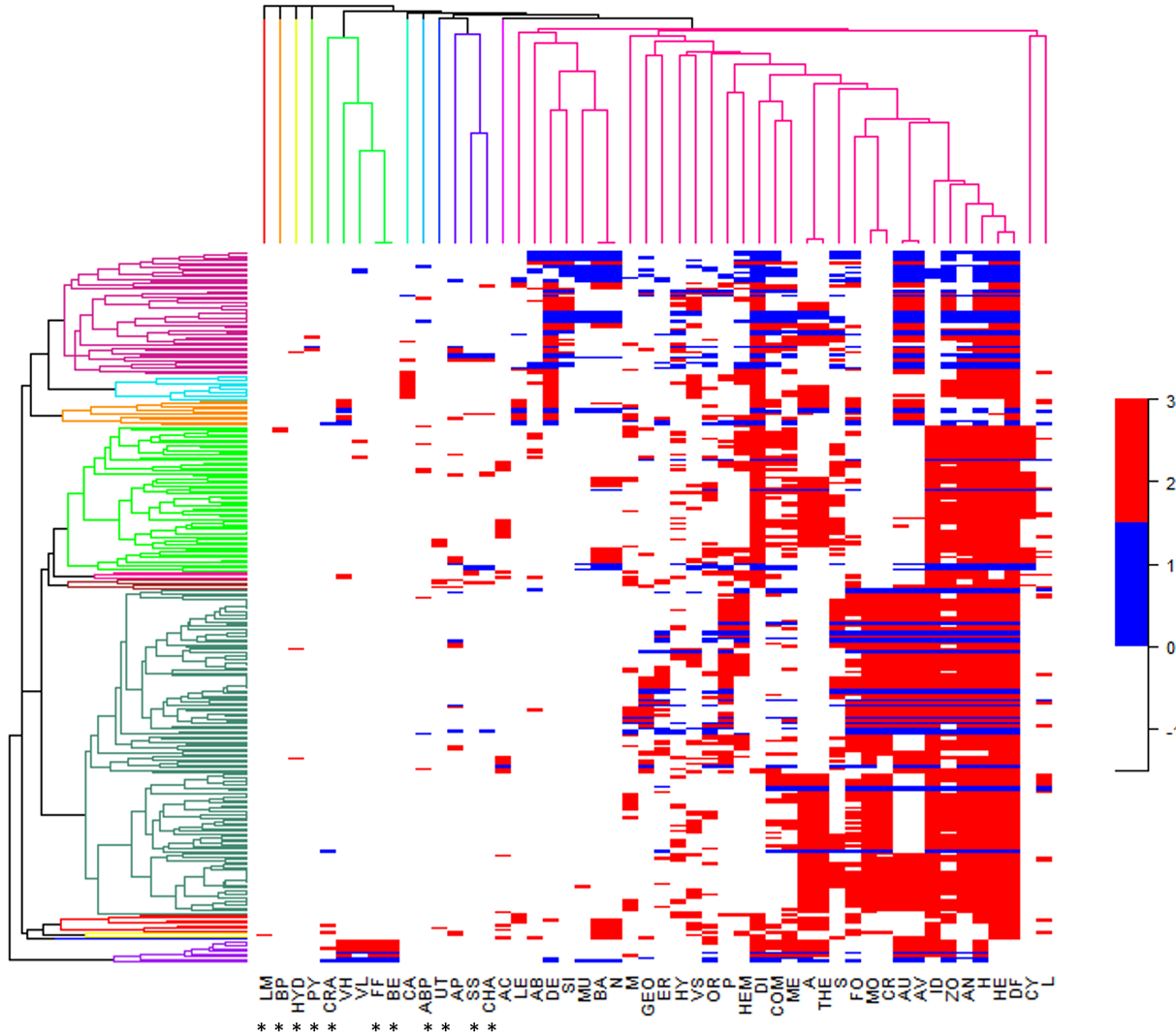

Supplement: Figure S1 — * Biological attributes and usages eliminated (did not contribute to the classification of NNS). [file peerj-06-5444-s003.png]
