# Supplementary material for: Darwin’s naturalization hypothesis does not explain the spread of nonnative weed species naturalized in México
Source: PeerJ. 2018 Aug 17;6:e5444. doi: 10.7717/peerj.5444 (PMC6100849; doi:10.7717/peerj.5444)
Supplement: Table S1 — *= Species with residence time smaller than 54 years. [file peerj-06-5444-s004.docx]

| **Num.** | **SPECIES** | **FAMILY** |
| --- | --- | --- |
| 1 | *Atriplex patula* L. | AMARANTHACEAE |
| 2 | *Atriplex rosea* L. | AMARANTHACEAE |
| 3 | *Atriplex semibaccata* R.Br. | AMARANTHACEAE |
| 4 | *Atriplex suberecta* I.Verd. | AMARANTHACEAE |
| 5 | *Bassia hyssopifolia* (Pall.) Kuntze | AMARANTHACEAE |
| 6 | *Beta vulgaris* L. | AMARANTHACEAE |
| 7 | *Chenopodium giganteum* D.Don | AMARANTHACEAE |
| 8 | *Chenopodium glaucum* L. | AMARANTHACEAE |
| 9 | *Chenopodium murale* L. | AMARANTHACEAE |
| 10 | *Kochia scoparia* (L.) Schrad. | AMARANTHACEAE |
| 11 | *Salsola tragu*s L. | AMARANTHACEAE |
| 12 | *Anthemis cotula* L. | ASTERACEAE |
| 13 | *Artemisia absinthium* L. | ASTERACEAE |
| 14 | *Bellis perennis* L. | ASTERACEAE |
| 15 | *Calendula officinalis* L. | ASTERACEAE |
| 16 | *Carthamus tinctorius* L. | ASTERACEAE |
| *17 | *Centaurea benedicta* (L.) L. | ASTERACEAE |
| 18 | *Centaurea melitensis* L. | ASTERACEAE |
| 19 | *Centaurea solstitialis* L. | ASTERACEAE |
| 20 | *Chrysanthemum coronarium* L. | ASTERACEAE |
| 21 | *Cichorium intybus* L. | ASTERACEAE |
| *22 | *Cirsium vulgare* (Savi) Ten. | ASTERACEAE |
| 23 | *Cotula australis* (Sieber ex Spreng.) Hook.f. | ASTERACEAE |
| 24 | *Cotula coronopifolia* L. | ASTERACEAE |
| 25 | *Cyanthillium cinereum* (L.) H. Rob. | ASTERACEAE |
| 26 | *Cynara cardunculus* L. | ASTERACEAE |
| 27 | *Dendranthema morifolium* (Ramat.) Tzvelev | ASTERACEAE |
| *28 | *Emilia fosbergii* Nicolson | ASTERACEAE |
| 29 | *Emilia sonchifolia* (L.) DC. | ASTERACEAE |
| 30 | *Guizotia abyssinica* (L.f.) Cass. | ASTERACEAE |
| 31 | *Gynura aurantiaca* Sch.Bip. ex Miq. | ASTERACEAE |
| 32 | *Hedypnois rhagadioloides* (L.) F.W.Schmidt | ASTERACEAE |
| 33 | *Helminthotheca echioides* (L.) Holub | ASTERACEAE |
| 34 | *Hypochaeris glabra* L. | ASTERACEAE |
| *35 | *Hypochaeris radicata* L. | ASTERACEAE |
| 36 | *Lactuca sativa* L. | ASTERACEAE |
| 37 | *Lactuca serriola* L. | ASTERACEAE |
| 38 | *Lapsana communis* L. | ASTERACEAE |
| 39 | *Laphangium luteoalbum* (L.) Tzvelev | ASTERACEAE |
| 40 | *Leucanthemum lacustre* (Brot.) Samp. | ASTERACEAE |
| 41 | *Leucanthemum vulgare* (Vaill.) Lam. | ASTERACEAE |
| 42 | *Matricaria* *matricarioides* (Less.) Porter | ASTERACEAE |
| *43 | *Rhaponticum repens* (L.) Hidalgo | ASTERACEAE |
| 44 | *Senecio inaequidens* DC. | ASTERACEAE |
| 45 | *Senecio vulgaris* L. | ASTERACEAE |
| 46 | *Silybum marianum* (L.) Gaertn. | ASTERACEAE |
| 47 | *Soliva anthemifolia* (Juss.) Sweet | ASTERACEAE |
| 48 | *Soliva sessilis* Ruiz & Pav. | ASTERACEAE |
| 49 | *Sonchus asper* (L.) Hill | ASTERACEAE |
| 50 | *Sonchus oleraceus* L. | ASTERACEAE |
| 51 | *Sonchus tenerrimus* L. | ASTERACEAE |
| 52 | *Tanacetum parthenium* (L.) Sch.Bip. | ASTERACEAE |
| 53 | *Taraxacum officinale* F.H. Wigg. | ASTERACEAE |
| 54 | *Tragopogon porrifolius* L. | ASTERACEAE |
| 55 | *Youngia japonica* (L.) DC. | ASTERACEAE |
| 56 | *Brassica juncea* (L.) Czern. | ASTERACEAE |
| 57 | *Brassica napus* L. | BRASSICACEAE |
| 58 | *Brassica nigra* (L.) W.D.J. Koch | BRASSICACEAE |
| 59 | *Brassica rapa* L*.* | BRASSICACEAE |
| 60 | *Brassica* *tournefortii* Gouan | BRASSICACEAE |
| 61 | *Cakile maritima* Scop. | BRASSICACEAE |
| 62 | *Camelina sativa* (L.) Crantz | BRASSICACEAE |
| 63 | *Capsella bursa pastoris* (L.) Medik. | BRASSICACEAE |
| 64 | *Cardamine hirsuta* L. | BRASSICACEAE |
| 65 | *Diplotaxis muralis* (L.) DC. | BRASSICACEAE |
| *66 | *Diplotaxis tenuifolia* (L.) DC. | BRASSICACEAE |
| 67 | *Eruca vesicaria* (L.) Cav. | BRASSICACEAE |
| 68 | *Erucastrum gallicum* (Willd.) O.E. Schulz | BRASSICACEAE |
| 69 | *Hirschfeldia incana* (L.) Lagr.-Foss. | BRASSICACEAE |
| 70 | *Hornungia procumbens* (L.) Hayek | BRASSICACEAE |
| 71 | *Iberis amara* L. | BRASSICACEAE |
| 72 | *Lepidium didymum* L. | BRASSICACEAE |
| 73 | *Lepidium draba* L. | BRASSICACEAE |
| 74 | *Lepidium latifolium* L. | BRASSICACEAE |
| 75 | *Lobularia maritima* (L.) Desv. | BRASSICACEAE |
| 76 | *Matthiola incana* (L.) W.T. Aiton | BRASSICACEAE |
| 77 | *Nasturtium officinale* R.Br. | BRASSICACEAE |
| 78 | *Raphanus raphanistrum* L. | BRASSICACEAE |
| 79 | *Raphanus sativus* L. | BRASSICACEAE |
| *80 | *Rapistrum rugosum* (L.) All. | BRASSICACEAE |
| *81 | *Rorippa dubia* (Pers.) H. Hara | BRASSICACEAE |
| 82 | *Rorippa palustris* (L.) Besser | BRASSICACEAE |
| 83 | *Sinapis alba* L. | BRASSICACEAE |
| 84 | *Sinapis arvensis* L. | BRASSICACEAE |
| 85 | *Sisymbrium altissimum* L. | BRASSICACEAE |
| 86 | *Sisymbrium irio* L. | BRASSICACEAE |
| 87 | *Sisymbrium officinale* (L.) Scop. | BRASSICACEAE |
| 88 | *Sisymbrium orientale* L. | BRASSICACEAE |
| 89 | *Thlaspi arvense* L. | BRASSICACEAE |
| 90 | *Cerastium glomeratum* Thuill. | BRASSICACEAE |
| *91 | *Herniaria cinerea* subsp. *cinerea* | CARYOPHYLLACEAE |
| *92 | *Polycarpon tetraphyllum* (L.) L. | CARYOPHYLLACEAE |
| 93 | *Saponaria officinalis* L. | CARYOPHYLLACEAE |
| 94 | *Scleranthus annuus* L. | CARYOPHYLLACEAE |
| 95 | *Silene gallica* L. | CARYOPHYLLACEAE |
| 96 | *Spergula arvensis* L. | CARYOPHYLLACEAE |
| *97 | *Spergularia bocconi* (Scheele) Asch. & Graebn. | CARYOPHYLLACEAE |
| 98 | *Stellaria graminea* L. | CARYOPHYLLACEAE |
| 99 | *Stellaria montioides* (Edgew. & Hook. f.) Ghaz. | CARYOPHYLLACEAE |
| *100 | *Tissa villosa* (Pers.) Britton | CARYOPHYLLACEAE |
| 101 | *Citrullus lanatus* (Thunb.) Matsum. & Nakai | CARYOPHYLLACEAE |
| 102 | *Cucumis anguria* L. | CUCURBITACEA |
| 103 | *Cucumis dipsaceus* Ehrenb. ex Spach | CUCURBITACEA |
| 104 | *Cucumis melo* L. | CUCURBITACEAE |
| 105 | *Cucumis sativus* L. | CUCURBITACEAE |
| 106 | *Lagenaria siceraria* (Molina) Standl. | CUCURBITACEAE |
| 107 | *Luffa cylindrica* (L.) M.Roem. | CUCURBITACEAE |
| *108 | *Momordica balsamina* L. | CUCURBITACEAE |
| 109 | *Momordica charantia* L. | CUCURBITACEAE |
| 110 | *Cyperus alternifolius* L. | CUCURBITACEAE |
| 111 | *Cyperus difformis* L. | CUCURBITACEAE |
| 112 | *Cyperus esculentus* L. | CYPERACEAE |
| 113 | *Cyperus involucratus* Rottb. | CYPERACEAE |
| 114 | *Cyperus iria* L. | CYPERACEAE |
| 115 | *Cyperus oxylepis* Nees ex Steud. | CYPERACEAE |
| 116 | *Cyperus rotundus* L. | CYPERACEAE |
| 117 | *Euphorbia lathyris* L. | CYPERACEAE |
| 118 | *Euphorbia peplus* L. | EUPHORBIACEAE |
| *119 | *Euphorbia terracina* L. | EUPHORBIACEAE |
| *120 | *Alysicarpus ovalifolius* (Schumach.) J.Léonard | EUPHORBIACEAE |
| 121 | *Alysicarpus vaginalis* (L.) DC. | FABACEAE |
| 122 | *Clitoria ternatea* L. | FABACEAE |
| 123 | *Crotalaria retusa* L. | FABACEAE |
| 124 | *Lablab purpureus* (L.) Sweet | FABACEAE |
| *125 | *Lathyrus latifolius* L. | FABACEAE |
| 126 | *Lathyrus tingitanus* L. | FABACEAE |
| 127 | *Medicago lupulina* L. | FABACEAE |
| 128 | *Medicago polymorpha* L. | FABACEAE |
| 129 | *Medicago sativa* L. | FABACEAE |
| 130 | *Melilotus albus* Medik. | FABACEAE |
| 131 | *Melilotus indicus* (L.) All. | FABACEAE |
| 132 | *Melilotus officinalis* (L.) Lam. | FABACEAE |
| 133 | *Pisum sativum* L. | FABACEAE |
| 134 | *Pueraria phaseoloides* (Roxb.) Benth. | FABACEAE |
| *135 | *Securigera varia* (L.) Lassen | FABACEAE |
| 136 | *Trifolium dubium* Sibth. | FABACEAE |
| 137 | *Trifolium pratense* L. | FABACEAE |
| 138 | *Trifolium repens* L. | FABACEAE |
| *139 | *Vicia faba* L. | FABACEAE |
| 140 | *Vicia sativa* L. | FABACEAE |
| 141 | *Vigna unguiculata* (L.) Walp. | FABACEAE |
| 142 | *Plantago lanceolata* L. | FABACEAE |
| 143 | *Plantago major* L. | PLANTAGINACEAE |
| *144 | *Plantago ovata* Forssk. | PLANTAGINACEAE |
| 145 | *Aegilops cylindrica* Host | PLANTAGINACEAE |
| *146 | *Aira caryophyllea* L. | POACEAE |
| 147 | *Anthoxanthum odoratum* L. | POACEAE |
| 148 | *Arthraxon hispidus* (Thunb.) Makino | POACEAE |
| 149 | *Avena barbata* Pott ex Link | POACEAE |
| 150 | *Avena fatua* L. | POACEAE |
| 151 | *Avena sativa* L. | POACEAE |
| 152 | *Bothriochloa ischaemum* (L.) Keng | POACEAE |
| 153 | *Bothriochloa pertusa* (L.) A.Camus | POACEAE |
| *154 | *Brachiaria distachya* (L.) Stapf | POACEAE |
| 155 | *Bracharia mutica* (Forssk.) Stapf. | POACEAE |
| 156 | *Brachiaria reptans* (L.) C.A.Gardner & C.E.Hubb. | POACEAE |
| 157 | *Briza minor* L. | POACEAE |
| 158 | *Bromus catharticus* Vahl | POACEAE |
| 159 | *Bromus diandrus* Roth | POACEAE |
| 160 | *Bromus hordeaceus* L. | POACEAE |
| 161 | *Bromus inermis* Leyss. | POACEAE |
| 162 | *Bromus japonicus* Houtt. | POACEAE |
| 163 | *Bromus madritensis* L. | POACEAE |
| 164 | *Bromus rigidus* Roth | POACEAE |
| 165 | *Bromus rubens* L. | POACEAE |
| *166 | *Bromus secalinus* L. | POACEAE |
| 167 | *Bromus tectorum* L. | POACEAE |
| 168 | *Cenchrus ciliaris* L. | POACEAE |
| 169 | *Chloris gayana* Kunth | POACEAE |
| *170 | *Chrysopogon zizanioides* (L.) Roberty | POACEAE |
| 171 | *Coix lacryma-jobi* L. | POACEAE |
| 172 | *Cortaderia selloana* (Schult. & Schult.f.) Asch. & Graebn. | POACEAE |
| *173 | *Crypsis vaginiflora* (Forssk.) Opiz | POACEAE |
| 174 | *Cymbopogon citratus* (DC.) Stapf | POACEAE |
| 175 | *Cynodon dactylon* (L.) Pers. | POACEAE |
| *176 | *Cynodon nlemfuensis* Vanderyst | POACEAE |
| *177 | *Cynodon plectostachyus* (K.Schum.) Pilg. | POACEAE |
| 178 | *Cyrtococcum trigonum* (Retz.) A.Camus | POACEAE |
| 179 | *Dactylis glomerata* L. | POACEAE |
| 180 | *Dactyloctenium aegyptium* (L.) Willd. | POACEAE |
| 181 | *Dichanthium annulatum* (Forssk.) Stapf | POACEAE |
| 182 | *Dichanthium aristatum* (Poir.) C.E.Hubb. | POACEAE |
| 183 | *Digitaria bicornis* (Lam.) Roem. & Schult. | POACEAE |
| 184 | *Digitaria ischaemum* (Schreb.) Muhl. | POACEAE |
| 185 | *Digitaria nuda* Schumach. | POACEAE |
| 186 | *Digitaria eriantha* Steud. | POACEAE |
| 187 | *Digitaria sanguinalis* (L.) Scop. | POACEAE |
| 188 | *Digitaria setigera* Roth | POACEAE |
| 189 | *Digitaria ternata* (A.Rich.) Stapf | POACEAE |
| 190 | *Digitaria velutina* (Forssk.) P.Beauv. | POACEAE |
| 191 | *Echinochloa crus-galli* (L.) P.Beauv. | POACEAE |
| 192 | *Echinochloa pyramidalis* (Lam.) Hitchc. & Chase | POACEAE |
| *193 | *Eleusine coracana* (L.) Gaertn. | POACEAE |
| 194 | *Eleusine indica* (L.) Gaertn. | POACEAE |
| 195 | *Eleusine multiflora* Hochst. ex A.Rich. | POACEAE |
| 196 | *Elymus repens* (L.) Gould | POACEAE |
| 197 | *Eragrostis atrovirens* (Desf.) Trin. ex Steud. | POACEAE |
| 198 | *Eragrostis barrelieri* Daveau | POACEAE |
| 199 | *Eragrostis cilianensis* (All.) Vignolo ex Janch. | POACEAE |
| 200 | *Eragrostis curvula* (Schrad.) Nees | POACEAE |
| 201 | *Eragrostis lehmanniana* Nees | POACEAE |
| 202 | *Eragrostis pilosa* (L.) P.Beauv. | POACEAE |
| 203 | *Eragrostis superba* Peyr. | POACEAE |
| 204 | *Eragrostis viscosa* (Retz.) Trin. | POACEAE |
| 205 | *Euclasta condylotricha* (Hochst. ex Steud.) Stapf | POACEAE |
| 206 | *Festuca arundinacea* Schreb. | POACEAE |
| 207 | *Festuca ovina* L. | POACEAE |
| 208 | *Gastridium ventricosum* (Gouan) Schinz & Thell. | POACEAE |
| 209 | *Glyceria fluitans* (L.) R.Br. | POACEAE |
| 210 | *Hackelochloa granularis* (L.) Kuntze | POACEAE |
| *211 | *Hainardia cylindrica* (Willd.) Greuter | POACEAE |
| 212 | *Hemarthria altissima* (Poir.) Stapf & C.E.Hubb. | POACEAE |
| 213 | *Holcus lanatus* L. | POACEAE |
| 214 | *Hordeum marinum* subsp*. gussoneanum* (Parl.) Thell. | POACEAE |
| 215 | *Hordeum murinum* L. | POACEAE |
| 216 | *Hordeum vulgare* L. | POACEAE |
| 217 | *Hyparrhenia hirta* (L.) Stapf | POACEAE |
| 218 | *Hyparrhenia rufa* (Nees) Stapf | POACEAE |
| 219 | *Hyperthelia dissoluta* (Nees ex Steud.) Clayton | POACEAE |
| 220 | *Ischaemum rugosum* Salisb. | POACEAE |
| 221 | *Lamarckia aurea* (L.) Moench | POACEAE |
| 222 | *Lolium multiflorum* Lam. | POACEAE |
| 223 | *Lolium perenne* L. | POACEAE |
| 224 | *Lolium temulentum* L. | POACEAE |
| 225 | *Panicum maximum* Jacq. | POACEAE |
| 226 | *Melinis minutiflora* P.Beauv. | POACEAE |
| 227 | *Melinis repens* (Willd.) Zizka | POACEAE |
| 228 | *Miscanthus sinensis* Andersson | POACEAE |
| 229 | *Panicum antidotale* Retz. | POACEAE |
| 230 | *Panicum miliaceum* L. | POACEAE |
| 231 | *Parapholis incurva* (L.) C.E.Hubb. | POACEAE |
| 232 | *Pennisetum clandestinum* Hochst. ex Chiov. | POACEAE |
| 233 | *Pennisetum glaucum* (L.) R.Br. | POACEAE |
| 234 | *Pennisetum purpureum* Schumach. | POACEAE |
| 235 | *Pennisetum setaceum* (Forssk.) Chiov. | POACEAE |
| 236 | *Pennisetum villosum* Fresen. | POACEAE |
| 237 | *Phalaris brachystachys* Link | POACEAE |
| 238 | *Phalaris canariensis* L. | POACEAE |
| 239 | *Phalaris minor* Retz. | POACEAE |
| 240 | *Phalaris paradoxa* L. | POACEAE |
| 241 | *Phleum pratense* L. | POACEAE |
| 242 | *Poa annua* L. | POACEAE |
| *243 | *Poa compressa* L. | POACEAE |
| 244 | *Poa nemoralis* L. | POACEAE |
| 245 | *Poa pratensis* L. | POACEAE |
| 246 | *Polypogon monspeliensis* (L.) Desf. | POACEAE |
| 247 | *Polypogon viridis* (Gouan) Breistr. | POACEAE |
| *248 | *Rottboellia cochinchinensis* (Lour.) Clayton | POACEAE |
| 249 | *Saccharum officinarum* L. | POACEAE |
| 250 | *Saccharum villosum* Steud. | POACEAE |
| 251 | *Schismus* *barbatus* (L.) Thell. | POACEAE |
| 252 | *Secale cereale* L. | POACEAE |
| 253 | *Setaria sphacelata* (Schumach.) Stapf & C.E.Hubb. ex M.B.Moss | POACEAE |
| 254 | *Setaria viridis* (L.) P.Beauv. | POACEAE |
| *255 | *Sorghum arundinaceum* (Desv.) Stapf | POACEAE |
| 256 | *Sorghum bicolor* (L.) Moench | POACEAE |
| 257 | *Sorghum halepense* (L.) Pers. | POACEAE |
| 258 | *Sorghum* X *almum* Parodi (pro sp.) | POACEAE |
| 259 | *Tragus berteronianus* Schult. | POACEAE |
| 260 | *Urochloa panicoides* P.Beauv. | POACEAE |
| 261 | *Vulpia bromoides* (L.) Gray | POACEAE |
| 262 | *Vulpia myuros* (L.) C.C.Gmel. | POACEAE |
| *263 | *Zoysia matrella* (L.) Merr. | POACEAE |
| 264 | *Fagopyrum esculentum* Moench | POLYGONACEAE |
| 265 | *Fallopia convolvulus* (L.) Á.Löve | POLYGONACEAE |
| 266 | *Muehlenbeckia complexa* (A. Cunn) Meisn. | POLYGONACEAE |
| *267 | *Persicaria hydropiper* (L.) Delarbre | POLYGONACEAE |
| 268 | *Persicaria lapathifolia* (L.) Delarbre | POLYGONACEAE |
| 269 | *Persicaria maculosa* Gray | POLYGONACEAE |
| 270 | *Polygonum arenastrum* Boreau | POLYGONACEAE |
| 271 | *Polygonum argyrocoleon* Steud. ex Kunze | POLYGONACEAE |
| 272 | *Polygonum aviculare* L. | POLYGONACEAE |
| 273 | *Rumex acetosella* L. | POLYGONACEAE |
| 274 | *Rumex conglomeratus* Murray | POLYGONACEAE |
| 275 | *Rumex crispus* L. | POLYGONACEAE |
| *276 | *Rumex maritimus* L. | POLYGONACEAE |
| 277 | *Rumex obtusifolius* L. | POLYGONACEAE |
| 278 | *Rumex pulcher* L. | POLYGONACEAE |
