# Supplementary material for: Darwin’s naturalization hypothesis does not explain the spread of nonnative weed species naturalized in México
Source: PeerJ. 2018 Aug 17;6:e5444. doi: 10.7717/peerj.5444 (PMC6100849; doi:10.7717/peerj.5444)
Supplement: Table S2 — *= Species with residence time smaller than 300 years. [file peerj-06-5444-s005.docx]

| **Num.** | **SPECIES** | **FAMILY** |
| --- | --- | --- |
| *1 | *Atriplex canescens* (Pursh) Nutt. | AMARANTHACEAE |
| *2 | *Casuarina cunninghamiana* Miq. | CASUARINACEAE |
| *3 | *Casuarina equisetifolia* L. | CASUARINACEAE |
| *4 | *Acalypha amentacea* Roxb. | EUPHORBIACEAE |
| *5 | *Acalypha hispida* Burm.f. | EUPHORBIACEAE |
| *6 | *Hevea brasiliensis* (Willd. ex A.Juss.) Müll.Arg. | EUPHORBIACEAE |
| *7 | *Ricinus communis* L. | EUPHORBIACEAE |
| *8 | *Acacia melanoxylon* R.Br. | FABACEAE |
| *9 | *Albizia lebbeck* (L.) Benth. | FABACEAE |
| *10 | *Bauhinia variegata* L. | FABACEAE |
| 11 | *Cajanus cajan* (L.) Huth | FABACEAE |
| 12 | *Cassia fistula* L. | FABACEAE |
| *13 | *Cassia javanica* L. | FABACEAE |
| *14 | *Cercis siliquastrum* L. | FABACEAE |
| *15 | *Crotalaria pallida* Aiton | FABACEAE |
| *16 | *Delonix regia* (Hook.) Raf. | FABACEAE |
| *17 | *Indigofera hirsuta* L. | FABACEAE |
| *18 | *Peltophorum pterocarpum* (DC.) K. Heyne | FABACEAE |
| *19 | *Senna didymobotrya* (Fresen.) H.S.Irwin & Barneby | FABACEAE |
| *20 | *Senna siamea* (Lam.) H.S.Irwin & Barneby | FABACEAE |
| *21 | *Sesbania grandiflora* (L.) Pers. | FABACEAE |
| *22 | *Spartium junceum* L. | FABACEAE |
| *23 | *Sutherlandia frutescens* (L.) R.Br. | FABACEAE |
| *24 | *Tamarindus indica* L. | FABACEAE |
| *25 | *Phyllanthus acidus* (L.) Skeels | PHYLLANTHACEAE |
| *26 | *Arundo donax* L. | POACEAE |
| *27 | *Bambusa vulgaris* Schrad. ex J.C. Wendl. | POACEAE |
